# Evaluation of the resistance to Chinese predominant races of *Puccinia triticina* and analysis of effective leaf rust resistance genes in wheat accessions from the U.S. National Plant Germplasm System

**DOI:** 10.3389/fpls.2022.1054673

**Published:** 2022-10-26

**Authors:** Lin Zhang, Xuefang Zhao, Jingxian Liu, Xiaolu Wang, Wenping Gong, Quanguo Zhang, Yuping Liu, Hongfei Yan, Qingfang Meng, Daqun Liu

**Affiliations:** ^1^ College of Plant Protection, Hebei Agricultural University, Technological Innovation Center for Biological Control of Crop Diseases and Insect Pests of Hebei Province, Baoding, China; ^2^ School of Landscape and Ecological Engineering, Hebei Engineering University, Handan, China; ^3^ College of Agronomy, Shandong Agricultural University, Tai'an, China; ^4^ Crop Research Institute, Shandong Academy of Agricultural Sciences, Shandong Wheat Technology Innovation Center, Jinan, China; ^5^ National Engineering Laboratory of Wheat and Maize, Shandong Wheat Technology Innovation Center, Jinan, China; ^6^ Key Laboratory of Wheat Biology and Genetic Improvement in the North HuangHuai River Valley of Ministry of Agriculture, Shandong Wheat Technology Innovation Center, Jinan, China; ^7^ Institute of Cereal and Oil Crops, Hebei Academy of Agriculture and Forestry Sciences, Shijiazhuang, China

**Keywords:** leaf rust, wheat accessions, resistance gene, molecular markers, races

## Abstract

*Puccinia triticina*, which is the causative agent of wheat leaf rust, is widely spread in China and most other wheat-planting countries around the globe. Cultivating resistant wheat cultivars is the most economical, effective, and environmentally friendly method for controlling leaf rust-caused yield damage. Exploring the source of resistance is very important in wheat resistance breeding programs. In order to explore more effective resistance sources for wheat leaf rust, the resistance of 112 wheat accessions introduced from the U.S. National Plant Germplasm System were identified using a mixture of pathogenic isolates of THTT, THTS, PHTT, THJT and THJS which are the most predominant races in China. As a result, all of these accessions showed high resistance at seedling stage, of which, ninety-nine accessions exhibited resistance at adult plant stage. Eleven molecular markers of eight effective leaf rust resistance genes in China were used to screen the 112 accessions. Seven effective leaf rust resistance genes *Lr9*, *Lr19*, *Lr24*, *Lr28*, *Lr29*, *Lr38* and *Lr45* were detected, except *Lr47*. Twenty-three accessions had only one of those seven effective leaf rust resistance gene. Eleven accessions carried *Lr24*+*Lr38*, and 7 accessions carried *Lr9*+*Lr24*+*Lr38*, *Lr24*+*Lr38*+*Lr45*, *Lr24*+*Lr29*+*Lr38* and *Lr19+Lr38*+*Lr45* respectively. The remaining seventy-one accessions had none of those eight effective leaf rust resistance genes. This study will provide theoretical guidance for rational utilization of these introduted wheat accessions directly or for breeding the resistant wheat cultivars.

## Introduction

Wheat leaf rust, caused by *Puccinia triticina* Erikss., is a serious fungal disease of wheat which occurs in the majority of wheat-growing regions worldwide, especially in North Africa, Southeast and Central Asia, Eastern Europe, North and South America (Bolton et al., 2008). In China, leaf rust is a common disease threatening wheat production, especially in the North China Plain, the Middle and Lower Reaches of the Yangtze River, Southwest and Northwest Regions (Liu and Chen, 2012). Varying on wheat cultivars and disease period, 7% to 30% yield loss can be encountered and even more than 50% in severe cases (Huerta-Espino et al., 2011). In recent years, the occurrence of wheat leaf rust has been in ascendancy as a result of varying climatic conditions as evident in 2008, 2009, 2012, 2013 and 2015 in the whole country or some regions (Zhang et al., 2015; Zhang et al., 2020a; Zhang et al., 2020b).

The most economical, effective and environment-friendly method to control leaf rust is to cultivate resistant cultivars (Pink, 2002). However, the variation of virulence and the emergence of new races of *P. triticina* always leads to loss of the effective resistance of wheat cultivars, especially which carried single leaf rust resistance genes and large-scale planted, and increase the potential risk of leaf rust epidemic on wheat (Zhang et al., 2015). The THTT, THTS, PHTT, THJT, THJS, PHJT, and PHTS were predominant races of *P. triticina* in China from 2011-2015, of which, THTT and PHTT were also the predominant races in India (Zhang et al., 2020a, b; Bhardwaj et al., 2019). Most wheat cultivars in the major wheat-growing regions such as Henan, Shandong and Hebei province are susceptible to these races (Zhang et al., 2017a; Zhang et al., 2017b; Zhang et al., 2020a; Zhang et al., 2020b). Previous studies also revealed that many of the major wheat cultivars in China carry a few leaf rust resistance genes, such as *Lr1*, *Lr16*, *Lr26*, and *Lr37*, which have lost their effective resistance (Zhang et al., 2017a; Zhang et al., 2017b; Zhang et al., 2020a; Zhang et al., 2020b). So, it is necessary to explore and utilize the effective wheat resistance resources for the breeding of new, sustainable, and durable resistant wheat cultivars.

Gene postulation and molecular marker-assisted selection(MAS) are the most commonly used methods for identification and analysis of wheat leaf rust resistance genes (Zhang et al., 2017a). Gene postulation is a method for presupposing and identifying leaf rust resistance genes in wheat cultivars. This method uses a set of wheat leaf rust resistance near-isogenic lines or single gene lines, but it is easily influenced by genetic background, environmental conditions and human factors (Hu et al., 2014). In addition, the different virulent races of *P. triticina* to the differential lines are the key factors for gene postulation. Therefore, the high-resistance wheat cultivars carrying effective leaf rust resistance genes cannot be analyzed by gene postulation methods due to the lack of corresponding virulent races for these genes. For example, so far, there are no virulent races against the leaf rust resistance genes *Lr9*, *Lr19*, *Lr24*, *Lr28* and *Lr38* in China and many parts of the world (Zhang et al., 2020a; Zhang et al., 2020b). So these genes cannot be postulated in the wheat cultivars by gene postulation. MAS can effectively track corresponding genes by using the molecular markers closely linked to the leaf rust resistance genes (Ding et al., 2010). Most of the leaf rust resistence gene markers had been developed and successfully applied to identify the known leaf rust resistance genes in wheat cultivars and molecular breeding for disease resistance (Bassi et al., 2015; Wang et al., 2016; Gebrewahid et al., 2017; Beukert et al., 2020; Wu et al., 2020). For instance, MAS has been successfully applied to practical commercial wheat breeding for rust resistance genes *Lr34* and *Yr36* (Miedaner and Korzun, 2012). Therefore, the gap created by gene postulation methods can be bridged by MAS methods, which has high efficiency for the identification of effective resistance genes contained in wheat cultivars.

There are abundant wheat germplasm resources (more than 49,000) preserved in the National Germplasm Bank of China. However, according to previous studies, the proportion of Chinese wheat cultivars with high resistance to leaf rust is relatively low by identifying the resistance of the main or core wheat breeding materials (lines) to leaf rust in different regions, and the majority of Chinese main wheat cultivars(lines) carry only a few leaf rust resistance genes which have lost their effectiveness (Ding et al., 2010; Shi et al., 2011; Zhao et al., 2013; Zhang et al., 2017a; Zhang et al., 2017b; Gao et al., 2019; Zhang et al., 2019a). For example, only 14 of 182 wheat cultivars(lines) in Huang-Huai-Hai river wheat region were resistant to leaf rust at seedling stage, and a few resistance genes, such as *Lr1*, *Lr26*, and *Lr37* which had lost their effectiveness in China, were detected in these tested cultivars(lines) (Gao et al., 2019). It is therefore demand-driven to increase the genetic resources of wheat leaf rust resistance and the appropriate supplement to the wheat parent material resource pool that can lay a resource foundation for the breeding of more resistance cultivars. In the previous study, we preliminarily identified the resistance of 359 introduced accessions from the United States National Plant Germplasm System at seedling stage, of which 112 resistant accessions were screened (Unpublished data). So this study aimed to further identify the resistance of these 112 wheat accessions to the Chinese predominant races of *P. triticina* and determine the effective leaf rust resistance genes by MAS, and provide new and excellent resistance sources for wheat resistance breeding program in China.

## Materials and methods

### Plant materials

One hundred and twelve wheat accessions used in this study were provided by Dr. Harold Bockelman, National Plant Germplasm System (NPGS), USDA-ARS, Aberdeen, Idaho, USA. The susceptible wheat Thatcher, Zhengzhou 5389 and 8 Thatcher near-isogenic lines with single resistance genes *Lr9*, *Lr19*, *Lr24*, *Lr28*, *Lr29*, *Lr38*, *Lr45* and *Lr47*, the effective resistance genes until now in China, were provided by Wheat Leaf Rust Research Center of Hebei Agricultural University.

### *Puccinia triticina* isolates

Five predominant races of *P. triticina*, THTT, THTS, PHTT, THJT and THJS were used in this study. These races were collected and identified by Wheat Leaf Rust Research Center of Hebei Agricultural University in China in 2015.

### Evaluation of resistance to leaf rust at seedling stage

In 2016 and 2017, 112 wheat accessions, Thatcher and Zhengzhou 5389 were planted in 30×16 cm plastic trays in the greenhouses of Hebei Agricultural University. Each line was represented by 5 to 10 seedlings. These wheat materials were inoculated with predominant *P. triticina* races as described by Zhang et al. (2020a). Urediniospores of five predominant races of *P. triticina* were mixed with talcum powder in a 1:10 proportion and subsequently bestrewed on the pre-moistened leaves of the experimental wheat seedling. The inoculated seedlings were then transferred to a closed humid container for incubation at 18 to 24^o^C in darkness for 16 to 24 h, and subsequently moved to a greenhouse at 20±5^o^C and a photoperiod regime of 12-14 h with fluorescent light supplementation. Evaluation of infection types (IT) were performed at 12 days post-inoculation (dpi) as described by Roelfs (1984) when the disease was fully developed on the susceptible control Thatcher and Zhengzhou 5389. The identification experiment were repeated at least three times.

### Validation of adult plant resistance in field

All wheat accessions were tested and evaluated for their resistance at adult plant stage in the field nurseries at Baoding in Hebei province in 2016 and 2017. In mid-October 2015 and 2016, seeds of each wheat accession were sown in single rows according to the standard of row spacing of 30 cm and length 2 m per line. The susceptible control Zhengzhou 5389 were sown adjacent to and around the test rows. The spore suspension was prepared by mixing equal amounts of urediniospores of five predominant races and adding Tween-80 at a final concentration of 1%. The spore suspension was then sprayed on the wheat plants in mid-April (Tillering stage) of 2016 and 2017. The inoculated seedlings were covered with plastic film overnight to moisturize them. Disease investigation was carried out when the disease was fully developed about middle of May (Filling stage) of 2016 and 2017. The infection types to the mixed races were identified and recorded as described by Roelfs (1984).

### DNA extraction and molecular marker detection

The genomic DNA of wheat accessions were extracted according to the modified CTAB method (Gill et al., 1991). Eleven STS and SCAR markers for eight effective leaf rust resistance genes in China, *viz*. *Lr9*, *Lr19*, *Lr24*, *Lr28*, *Lr29*, *Lr38*, *Lr45* and *Lr47*, were used to screen the identified resistant wheat accessions (**Table 1**). PCR procedure was performed as described by references in **Table 1**. PCR products were detected by 1.0% (w/v) agarose gel electrophoresis in 1×TAE buffer and visualized under UV transilluminator.

**Table 1:** Primers of molecular markers used to detect the wheat leaf rust resistance.

*Lr* gene	Marker type	Primer name	Sequence of primer (5'-3')	FragmentSize (bp)	Reference
*Lr9*	SCAR	SCS5-550F	TGCGCCTTCAAAGGAAG	550	Gupta et al., 2005
		SCS5-550R	TGCGCCCTTCTGAACTGTAT		
*Lr9*	STS	J13/1	TCCTTTTATTCCGCACGCCGG	1100	Schachermayr et al., 1994
		J13/2	CCACACTACCCCAAAGAGACG		
*Lr19*	SCAR	SCS265-F	GGCGGATAAGCAGAGCAGAG	512	Gupta et al., 2006a
		SCS265-R	GGCGGATAAGTGGGTTATGG		
*Lr19*	SCAR	SCS253-F	GCTGGTTCCACAAAGCAAA	736	Gupta et al., 2006a
		SCS253-R	GGCTGGTTCCTTAGATAGGTG		
*Lr24*	STS	J09/1	TCTAGTCTGTACATGGGGGC	310	Schachermayr et al., 1995
		J09/2	TGGCACATGAACTCCATACG		
*Lr24*	SCAR	S1302_609_-F	CGCAGGTTCCAAATACTTTTC	607	Gupta et al., 2006b
		S1302_609_-R	CGCAGGTTCTACCTAATGCAA		
*Lr28*	SCAR	SCS421_570_-F	ACAAGGTAAGTCTCCAACCA	570	Cherukuri et al., 2005
		SCS421_570_-R	AGTCGACCGAGATTTTAACC		
*Lr29*	SCAR	OPY10/1	GTGACCTCAGGCAATGCA	850	Tar et al., 2002
		OPY10/2	GTGACCTCAGAACCGATG		
*Lr38*	SCAR	Y_38_SCAR_982_-F	GCTGAATCTGCGTATCGTCCC	982	Yan et al., 2008
		Y_38_SCAR_982_-R	GACTTGTTCTTCGGCGTGTTG		
*Lr45*	SCAR	PSc20H23	CGACGATCGAATCT CGGGCAAG	750	Yan, 2009
		PSc20H24	GCGCCCTGCGTTGAGGAGAC		
*Lr47*	STS	PS10R	GCTGATGACCCTGACCGGT	282	Helguera et al., 2000
		PS10L	TCTTCATGCCCGGTCGGGT		

## Results

### Seedling resistance

In this study, the predominant races THTT, THTS, PHTT, THJT and THJS were used to identify the leaf rust resistance of 112 wheat accessions at seedling stage. The identification results showed that these wheat accessions showed different degrees of resistance to leaf rust at seedling stage (**Table 2**). Seven out of the 112 wheat accession representing 6.25 % of the total accessions (PI601428, PI542975, PI601429, PI478892, PI639450, Citr15929, and Citr15082) exhibited immunity (IT “0”). Sixty-eight accessions showed high resistance with ITs “;” or “1”, while 37 other accessions showed moderate resistance with ITs “X”, such as “;1, 3” or “;, 3”. These results indicated higher resistance rates of these wheat cultivars to Chinese *P. triticinia* race. The wheat accessions with ITs “X” may be due to the specific resistance to some of the isolates of *P. triticinia*.

**Table 2:** Leaf rust resistance levels at seedling and adult stages and detection results of molecular markers.

No.	accessions	Seeding Infection type	Adult Infection type	*Lr* gene	No.	accessions	Seeding Infection type	Adult Infection type	*Lr* gene
1	PI 601428	0	;1	*Lr24, Lr38*	57	CItr 17723	;, 1	;1	*Lr29*
2	PI 601429	0	;	*Lr9*	58*	CItr 17831	;1, 3	;	*—*
3	PI 601465	;	;	*Lr9*	59	CItr 17856	;, 3	;1, 3	—
4	PI 601606	1	;1	*Lr24, Lr38*	60	CItr 17857	;1, 3	;1, 3	*—*
5	PI 595212	;	;	—	61	CItr 15075	;1	;1, 3	—
6	PI 17389	;1, 3	;1, 3	*—*	62	CItr 15082	0	;	*Lr9*
7	PI 17879	;1	;1	*—*	63	CItr 15290	;1	;	—
8	PI 17898	;1	;	—	64	PI 548844	;	;	*Lr9*
9	PI 486147	;	;	*Lr24, Lr38*	65	PI 548845	;1, 3	;1, 3	—
10	PI 486212	;	;	*Lr24, Lr38*	66	PI 548847	;	;	—
11*	PI 486349	;1, 3	;	—	67	PI 550697	;1, 3	;1, 3	—
12	PI 494101	;1	;	—	68	PI 552813	;	;1, 3	*Lr45*
13	PI 542975	0	;1, 3	—	69	PI 543893	;	;	*Lr24, Lr38, Lr45*
14	PI 542979	;	;	*Lr9, Lr24, Lr38*	70	PI 547262	;1	;1	—
15	PI 547264	;	;1	*Lr45*	71*	PI 547263	1, 3	;	—
16	PI 594102	1	;, 3	—	72	PI 555586	;	;1, 3	*—*
17	PI 497988	;	;1	*—*	73*	CItr 3780	;, 3	;1	*—*
18	PI 17729	;	;1	*—*	74	CItr 15375	;	;	*Lr9*
19	PI 601262	;1	;	—	75	CItr 17264	;1, 3	;1, 3	—
20	PI 601263	;	;	*Lr9*	76	PI 564700	;	;1, 3	—
21	PI 601366	;	;	—	77	PI 564851	;	;	*Lr24, Lr29, Lr38*
22	PI 601207	;	;	—	78	PI 566923	;1	;1	*Lr24*
23	PI 601203	;1	;	*Lr24, Lr38*	79	PI 577793	;	;	*Lr24, Lr38*
24	PI 598214	;	;1	—	80	PI 578213	;1	;	*Lr24, Lr38, Lr45*
25	PI 599987	1	;	—	81	PI 491396	;	;	*Lr9*
26*	PI 598209	;1, 3	;	—	82	PI 583676	;1	;1	*Lr29*
27	PI 598211	;1	;	—	83	PI 591560	;1	;1	—
28	PI 598212	;	;	—	84	PI 476974	;1	;1	*Lr29*
29*	PI 298213	;1, 3	;1	—	85	PI 476975	;1	;	*Lr28*
30	PI 486140	;1	;	*Lr45*	86	PI 483469	;1	;	*Lr24, Lr38, Lr45*
31*	PI 508288	;1, 3	;	*—*	87	PI 596335	;1	;	*Lr24, Lr38, Lr45*
32	PI 511307	;1	;1	*Lr24, Lr38*	88*	PI 601722	;1, 3	;	*—*
33	PI 511308	;1	;	*Lr24, Lr38*	89	PI 559376	;	;	*—*
34	PI 506407	;	;	*Lr24, Lr38*	90	PI 557537	;1	;	*Lr28*
35	PI 506405	;	;	*Lr29*	91	PI 557538	;1, 3	;1, 3	*—*
36	PI 531246	;	;	*Lr24, Lr38*	92	PI 561197	;	;1	*—*
37	Citr 17940	;1	;	*—*	93*	PI 561198	;1, 3	;1	*—*
38	PI 600974	;1, 3	;, 3	—	94	PI 641952	1	;1	*—*
39*	PI 601069	1, 3	;	—	95*	PI 561200	;1, 3	;	*—*
40*	PI 601070	;1, 3	;	*—*	96*	PI 562382	1^+^	;	*—*
41	PI 601723	;	;	*Lr29*	97	PI 639450	0	;1	*Lr45*
42	PI 601806	;1	;1	*Lr29*	98	PI 564083	;1, 3	;1, 3	*—*
43	PI 601807	;1	;1	*—*	99*	PI 573003	;1, 3	;	*—*
44	Citr 13684	;1	;1	*—*	100	PI 601452	;1, 3	3	*—*
45	Citr 13874	;	;1, 3	*—*	101	PI 591702	;1, 3	3	*—*
46	Citr 14048	;	;	*Lr19, Lr38, Lr45*	102	PI 486145	;1, 3	3	*—*
47	Citr 15229	;	;	*Lr45*	103	PI 542976	;1, 3	3	*—*
48	Citr 15288	;	;	*Lr9*	104	PI 547082	;1, 3	3	*—*
49	Citr 15929	0	;	*Lr9*	105	PI 17769	;1, 3	3	*—*
50	Citr 17262	;	;1	*—*	106	PI 478892	0	3	*—*
51*	PI 535454	;1, 3	;1	*—*	107	PI 516197	;1, 3	3	*—*
52	PI 518591	;	;	—	108	PI 531197	;1, 3	3	*—*
53*	PI 527480	;, 3	;	—	109	PI 468977	;1, 3	3	*—*
54	PI 531244	;	;1, 3	*—*	110	PI 469272	;1, 3	3	*—*
55	PI 532282	1	;	*Lr24, Lr38*	111	PI 475771	;1	3	*—*
56*	PI 532912	;1, 3	;	*—*	112	PI 566924	;1, 3	3	*—*

“0”: No chlorotic flecks or uredinia; “;”: No uredinia, but flecks or chlorosis; “1”: Small uredinia with necrosis; “3”: Moderate size uredinia with slight chlorosis; “+”: uredinia somewhat larger than normal for the infection type; “—”: No tested gene is detected. “*”: wheat accessions with higher resistance at adult plant stage than at seedling stage.

### Field resistance

To further characterize the resistance of 112 wheat accessions to leaf rust at adult plant stage, field nursery experiments were carried out in the wheat cropping seasons. Ninety-nine (99) of 112 wheat accessions were resistant at adult plant stage, which were consistent with the seedling stage (**Table 2**). The remaining 13 accessions (PI601452, PI591702, PI486145, PI542976, PI547082, PI17769, PI478892, PI516197, PI531197, PI468977, PI469272, PI475771, and PI566924) were susceptible to leaf rust at adult plant stage with ITs “3” indicating that the seedling resistance, so-called whole growth period resistance, may encountered a new phenotype. In addition, 17 wheat accessions (marked with asterisks in **Table 2**) with ITs “X” to leaf rust at seedling stage conferred higher resistance at adult plant stage, implying that those accessions may carry adult leaf rust resistance genes or the heat sensitive resistance gene which induced the resistance to *P. triticinia* at high temperature.

### Detection of resistance genes

**Figure 1:**
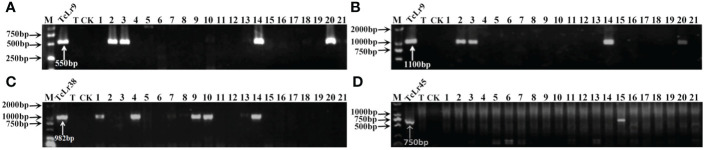
PCR amplifications results of molecular markers SCS5-550F/R **(A)** and J131/2 **(B)** for *Lr9*, Y_38_SCAR_982_-F/R for *Lr38* **(C)**, and PSc20H23/24 for *Lr45* **(D)** in part of accessions. M, DL2000 Marker; TcLr9, TcLr38 and TcLr45: Thatcher near-isogenic lines with single resistance genes *Lr9*, *Lr38* and *Lr45* (Positive control); T, Thatcher (Negative control); CK, ddH_2_O, Lane 1-21: PI 601428, PI 601429, PI 601465, PI 601606, PI 595212, PI 17389, PI 17879, PI 17898, PI 486147, PI 486212, PI 486349, PI 494101, PI 542975, PI 542979, PI 547246, PI 594102, PI 497988, PI 17729, PI 601262, PI 601263, PI 601366.

**Figure 2:**
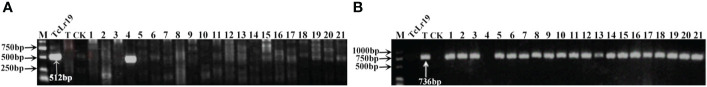
PCR amplifications results of molecular markers SCS265-F/R(Coupling) **(A)** and SCS253-F/R(Repulsion) **(B)** for *Lr19* in part of accessions. M, DL2000 Marker; TcLr19, Thatcher near-isogenic lines with single resistance genes *Lr19* (Positive control); T, Thatcher (Negative control); CK, ddH2O, Lane 1-21, PI 601807, Citr 13684, Citr 13874, Citr 14048, Citr 15229, Citr 15288, Citr 15929, Citr 17262, PI 535454, PI 518591, PI 527480, PI 531244, PI 532282, PI 532912, CItr 17723, CItr 17831, CItr 17856, CItr 17857, CItr 15075, CItr 15082, CItr 15290.

**Figure 3:**
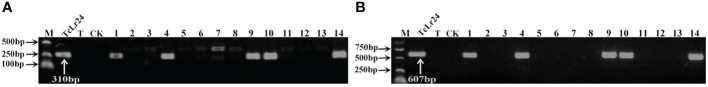
PCR amplifications results of molecular markers J09/1/2 **(A)** and S1302_609_-F/R **(B)** for *Lr24* in part of accessions. M, DL2000 Marker; TcLr24, Thatcher near-isogenic lines with single resistance genes *Lr24* (Positive control); T, Thatcher (Negative control); CK, ddH2O, Lane 1-14, PI 601428, PI 601429, PI 601465, PI 601606, PI 595212, PI 17389, PI 17879, PI 17898, PI 486147, PI 486212, PI 486349, PI 494101, PI 542975, PI 542979.

**Figure 4:**
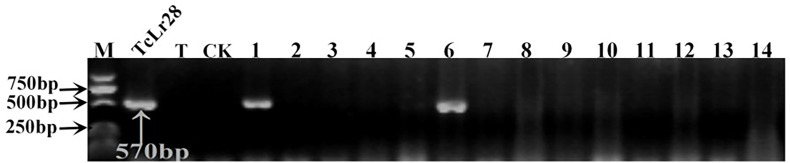
PCR amplifications results of molecular markers SCS421_570_-F/R for *Lr28* in part of accessions. M, DL2000 Marker; TcLr28, Thatcher near-isogenic lines with single resistance genes *Lr28* (Positive control); T, Thatcher (Negative control); CK, ddH2O, Lane 1-14, PI 476975, PI 483469, PI 596335, PI 601722, PI 559376, PI 557537, PI 557538, PI 561197, PI 561198, PI 641952, PI 561200, PI 562382, PI 639450, PI 564083.

**Figure 5:**
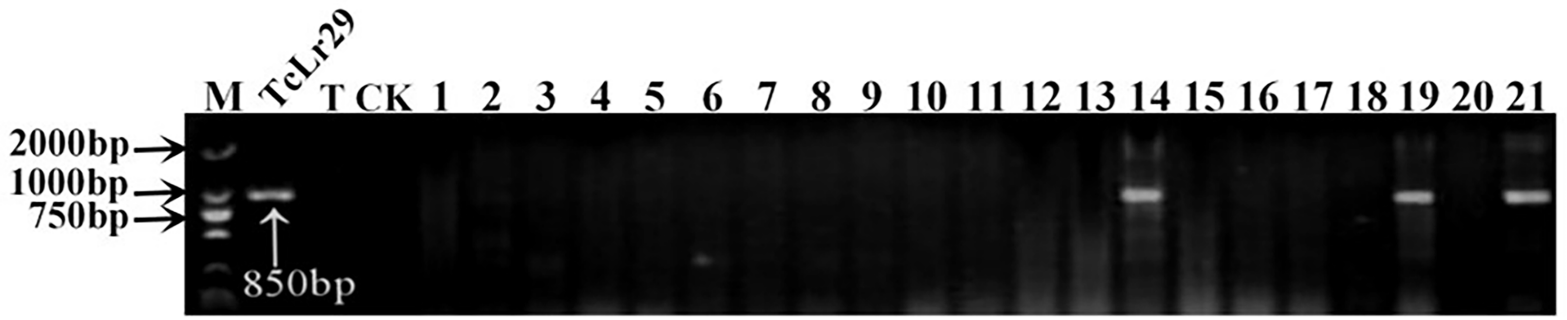
PCR amplifications results of molecular markers OPY10/1/2 for *Lr29* in part of accessions. M, DL2000 Marker; TcLr29, Thatcher near-isogenic lines with single resistance genes *Lr29* (Positive control); T, Thatcher (Negative control); CK, ddH2O, Lane 1-21, PI 548844, PI 548845, PI 548847, PI 550697, PI 552813, PI 543893, PI 547262, PI 547263, PI 555586, CItr 3780, CItr 15375, CItr 17264, PI 564700, PI 564851, PI 566923, PI 577793, PI 578213, PI 491396, PI 583676, PI 591560, PI 476974.

To further identify the leaf rust resistance genes carried by these wheat accessions, the STS and SCAR markers of eight effective leaf rust resistance genes in China were used to detect the leaf rust resistance genes of these accessions. Seven leaf rust resistance genes, *Lr9*, *Lr19*, *Lr24*, *Lr28*, *Lr29*, *Lr38* and *Lr45*, were detected in 41 of 112 wheat accessions (**Figure 1**–**5**, **Table 2**). No corresponding leaf rust resistance genes were detected in the remaining 71 accessions, indicating that other unknown or new effective leaf rust resistance genes at seedling stage existed in these resistant accessions. Based on marker analyses, the 41 resistant accessions carrying the tested gene can be divided into three categories: The first type consisted of wheat accessions that carried only a single leaf rust resistance gene. It was observed that, 9 accessions carried *Lr9*, one accession carried *Lr24*, two accessions carried *Lr28*, 6 accessions carried *Lr29*, and 5 accessions carried *Lr45*, which accounted for 22.0%, 2.4%, 4.9%, 14.6% and 12.2% of 41 resistant accessions respectively. The second type was made up of *Lr24* and *Lr38* which existed in 11 wheat accessions representing 26.8%. The third type: *Lr9*+*Lr24*+*Lr38* were detected in 1 accession, *Lr24*+*Lr38*+*Lr45* in 4 accessions, *Lr24*+*Lr29*+*Lr38* in 1 accession, and *Lr19*+*Lr38*+*Lr45* in 1 accession, which respectively accounted for 2.4%, 9.8%, 2.4% and 2.4% of 41 resistant accessions. The remaining 58 materials carried unknown effective leaf rust resistance genes, which accounted for 58.6% of 99 accessions with whole growth period resistance. These results indicated that the utilization ratios of *Lr45*, *Lr24* and *Lr38* were the highest among these accessions, with *Lr45* accounted for 10.1%, *Lr24* for 18.2% and *Lr38* for 18.2% in 99 resistant accessions. In addition, *Lr47* was not detected in any of the tested wheat accessions in this study.

## Discussion

Races of *P. triticina*, especially the predominant races THTT, THTS, PHTT, THJT and THJS from the wheat-growing regions of China, posed serious threat to wheat production in 2011-2015 due to high virulence to many cultivars and their widespread distribution (Zhang et al., 2020a; Zhang et al., 2020b). According to the field investigation and the identification of resistance to leaf rust, the majority of main wheat cultivars in the main wheat-growing regions were susceptible to leaf rust in China. For instance, at least 28 main wheat cultivars cultivated in the North China Plain, where is the largest wheat wheat-growing region with the highest wheat yield, were found to be susceptible to wheat leaf rust in recent years (Zhang et al., 2017a; Zhang et al., 2017b; Zhang et al., 2019b). Most of the Chinese wheat cultivars, including the above mentioned, carried a few leaf rust resistance genes such as *Lr*1, *Lr16*, *Lr26*, *Lr37* among others (Gebrewahid et al., 2017; Zhang et al., 2017a; Zhang et al., 2017b; Gao et al., 2019; Zhang et al., 2019). Among these genes, *Lr1* and *Lr26* were the most used leaf rust resistance gene(s) in China. The proportions of *Lr1* and *Lr26* in 460 Chinese wheat accessions were 47.8% and 33.5% respectively (Zhang, 2015), while that of *Lr26* in 116 different wheat accessions in a study by Ren (2011) was observed to be as high as 37 %. While these genes have lost their effectiveness (Zhang et al., 2020a; Zhang et al., 2020b), which is key reasons for the poor resistance of wheat cultivars to leaf rust in China, so it is necessary to screen and identify more new sources of leaf rust resistance genes.

Wheat cultivars introduced from USA may confer different resistance sources compared with Chinese common wheat cultivars, due to the *P. triticina* predominant populations and the frequencies of virulence to leaf rust resistance genes are different (Kolmer and Hughes, 2017; Kolmer, 2019; Zhang et al., 2020a; Zhang et al., 2020b). For example, the wheat cultivars with the resistance genes *Lr9*, *Lr21*, *Lr24*, and *Lr39* have been released since the 1960s-1980s in the United States (Huerta-Espino et al., 2008; Kolmer et al., 2018), but these genes are rarely used in Chinese wheat cultivars. Some of these leaf rust resistance genes had lost their effectiveness, for instance, *Lr24* in the United State has begun to lose effectiveness to *P. triticinia* (Kolmer, 2019), but it is known to confer effective resistance in China and until now the virulent race of *P. triticinia* to *Lr24* is not be found. Against above background, we used the predominant races of Chinese *P. triticina* to identify the resistance of wheat cultivars from the United States for better and faster application in breeding. Due to the problem of hybridization incompatibility, it is more advantageous to select wheat resistant materials as parents for hybridization breeding compared with wild relatives of wheat or foreign gene introduction. Therefore, it is necessary to search for effective leaf rust resistance genes in known wheat cultivars or lines, especially those introduced cultivars which may have new potential resistance sources. In this study, 112 wheat accessions from the United States were resistant to Chinese predominant races of *P. triticina* at seedling stage, which indicated that the resistance resources of these wheat materials in the United States were abundant and may be a good source of resistance against wheat leaf rust in China. Moreover, the resistance of 99 out of the 112 wheat accessions also exhibited resistance to *P. triticinia* at adult plant stage, suggesting these accessions confers whole growth period resistance to leaf rust from seedling stage to adult plant stage. These cultivars were therefore subjected to effective leaf rust resistance gene analysis using molecular makers.

At present, 82 leaf rust resistance genes have been given gene designations (Bariana et al., 2022). The leaf rust resistance genes such as *Lr1*, *Lr2a*, *Lr2c*, *Lr3*, *Lr16*, *Lr26*, *Lr11*, *Lr17*, *LrB*, *Lr10*, *Lr14a*, *Lr2b*, *Lr3bg*, *Lr14b*, *Lr32*, *Lr33*, *Lr37*, and *Lr50* have lost the effectiveness in China from 2011 to 2015 (Zhang et al., 2020a; Zhang et al., 2020b). A few leaf rust resistance genes such as *Lr9*, *Lr19*, *Lr24*, *Lr28*, *Lr29*, *Lr38*, *Lr45* and *Lr47* still possessed their effective resistance to inhibit most of *P. triticina* isolates including those predominant races as mentioned above in China (Zhang et al., 2020a; Zhang et al., 2020b). These leaf rust resistance genes express effective resistance at both seedling and adult plant stages. No or very few race have been found to be virulent to these effective leaf rust resistance genes in China, so gene postulation method was difficult to be used for the analysis of these genes. While the molecular marker-assisted selection method is preferred and convenient for leaf rust resistance genes detection because of its rapidity and accuracy (Ding et al., 2010). Seven effective leaf rust resistance genes, *Lr9*, *Lr19*, *Lr24*, *Lr28*, *Lr29*, *Lr38* and *Lr45*, were detected in 41 of these accessions, which similar as the research reports that wheat cultivars with the leaf rust resistance genes *Lr9*, *Lr21*, *Lr24*, and *Lr39* have been released since the 1960s-1980s in the United States (Huerta-Espino et al., 2008; Kolmer et al., 2018). The exception to this assertion was *Lr21* and *Lr39* genes. The resistance of *Lr21* and *Lr39* to the Chinese predominant races of *P. triticina* were relatively low due to fact that they were losing their effectiveness (Zhang et al., 2020a; Zhang et al., 2020b). Research findings on the identification of wheat leaf rust resistance resources in China revealed that these effective leaf rust resistance genes are rarely distributed and accounted for a very low proportion in the wheat cultivars(lines) that have been in cultivation in China (Gao et al., 2019; Ren et al., 2012; Shi, 2010; Zhang, 2015). The tested leaf rust resistance genes were not be detected in some resistance accessions by the known leaf rust resistance gene markers, the main reason should be due to absent the correspondence resistance genes or maybe unknown leaf rust resistance gene in these wheat accessions. Although these tested effective leaf rust resistance genes were not present in the remaining 71 resistant accessions in this study, their high resistance phenotype indicated that these wheat accessions may carry others undetected, known or new leaf rust resistance genes. Therefore, all these resistant cultivars have certain potential as breeding materials in China.

In general, leaf rust resistance genes are broadly divided into two main categories: seedling resistance genes and adult plant resistance genes (Riaz, 2018). Most of the designated leaf rust resistance genes are the seedling resistance genes. These genes are usually detected at the seedling stage and remain effective throughout the growth stages of wheat. These genes are therefore known as all-stage resistance genes. Adult plant resistance genes are usually effective at the post-seedling stage. At present, among the designated 82 leaf rust resistance genes, only 16, *Lr12*, *Lr13*, *Lr22* (alleles a, and b), *Lr34*, *Lr35*, *Lr37*, *Lr46*, *Lr48*, *Lr49*, *Lr67*, *Lr68*, *Lr74*, *Lr75*, *Lr77* and *Lr78* specifically provide resistance at the adult plant stage (Kolmer et al., 2017). Some adult plant resistance genes are also known as slow rusting genes, such as *Lr34*, *Lr46*, *Lr67*, and *Lr68* because they can confer partial resistance or slow rusting resistance (Parlevliet and Ommeren, 1985; Zhang et al., 2019). Thirteen of the 112 seedling resistance accessions were susceptible at adult plant stage which might have been caused by the temperature-sensitive genes. For example, the known temperature-sensitive leaf rust resistance genes *Lr11*, *Lr14a*, *Lr14b*, *Lr18*, *Lr20* and *Lr37* were noted to be more effective at lower temperatures (Mcintosh et al., 1995; Zhang et al., 2008; Wang et al., 2016). This phenomenon is worth further verification for conclusive establishment. Seventeen wheat accessions (marked with asterisks in **Table 2**) with moderate resistance at seedling stage exhibited high resistance at adult plant stage which indicated these wheat accessions may also carry polymeric genes with adult plant resistance gene (Kolmer, 2019).

The long-term cultivation of single resistance gene cultivars, especially those with single resistance gene cultivars are easy to lose resistance to leaf rust, while wheat cultivars with multiple resistance genes are more durable (Mcintosh, 1992). In this study, 18.2% of the resistant accessions carried polymeric genes, mainly including five types of polymeric genes, *Lr24*+*Lr38*, *Lr9*+*Lr24*+*Lr38*, *Lr24*+*Lr38*+*Lr45*, *Lr24*+*Lr29*+*Lr38* and *Lr19*+*Lr38*+*Lr45*, among which the wheat accessions that carried polymeric genes *Lr24*+*Lr38* were the most numerous. These wheat accessions showed high resistance at both seedling stage and adult plant stage, especially those accessions with polymeric genes were very valuable for breeding cultivars with resistance throughout the whole growth period. The rational utilization of polymeric genes can inhibit the predominant virulence race, stabilize the pathogen population by directional selection, and thus reduce the incidence and epidemic of leaf rust disease, and make the resistance of cultivars more durable (Staskawicz et al., 1995). Therefore, it is the future trend of wheat breeding to select polymeric leaf rust resistance genes with effectiveness, high resistance, and good comprehensive traits. In addition, the balance between yield traits and resistance traits is also important, and we need to pay attention to the coordination between them. In order to improve the level of leaf rust resistance of wheat cultivars, it is necessary for us to pyramid those effective leaf rust resistance genes into new cultivars without affecting other agronomic traits.

## Conclusion

In the present study, we identified the seedling and adult plant resistance of 112 wheat accessions introduced from the U.S. National Plant Germplasm System using a mixture of Chinese predominant *P. triticinia* races THTT, THTS, PHTT, THJT and THJS. Seven effective resistance genes *Lr9*, *Lr19*, *Lr24*, *Lr28*, *Lr29*, *Lr38* and *Lr45* singly or in combination were found in 41 wheat accessions. Our study will provide theoretical guidance for rational using some of these wheat accessions as resistance material or variety to breeding program.

## Data availability statement

The original contributions presented in the study are included in the article. Further inquiries can be directed to the corresponding authors.

## Author contributions

HY, QM and DL designed the experiments. LZ and XZ carried out the experiments and wrote the manuscript. JL, XW, WG, QZ, and YL participated or assisted in some part of the study. All authors contributed to the article and approved the submitted version.

## Funding

This research was supported by Hebei Province Wheat Industry System (Grant No. HBCT2018010204), Key Research and Development Program of Hebei (Grant No. 21326508D).

## Acknowledgments

We thank Dr. Harold Bockelman from National Plant Germplasm System (NPGS), USDA-ARS, Aberdeen, Idaho, USA for providing the wheat accessions.

## Conflict of interest

The authors declare that the research was conducted in the absence of any commercial or financial relationships that could be construed as a potential conflict of interest.

## Publisher’s note

All claims expressed in this article are solely those of the authors and do not necessarily represent those of their affiliated organizations, or those of the publisher, the editors and the reviewers. Any product that may be evaluated in this article, or claim that may be made by its manufacturer, is not guaranteed or endorsed by the publisher.
